# Plant neighbour-modulated susceptibility to pathogens in intraspecific mixtures

**DOI:** 10.1093/jxb/erab277

**Published:** 2021-06-14

**Authors:** Rémi Pélissier, Luis Buendia, Andy Brousse, Coline Temple, Elsa Ballini, Florian Fort, Cyrille Violle, Jean-Benoit Morel

**Affiliations:** 1 PHIM Plant Health Institute, Université de Montpellier, Institut Agro, CIRAD, INRAE, IRD, Montpellier, France; 2 PHIM Plant Health Institute, Université de Montpellier, CIRAD, INRAE, Institut Agro, IRD, Montpellier, France; 3 PHIM Plant Health Institute, Université de Montpellier, INRAE, CIRAD, Institut Agro, IRD, Montpellier, France; 4 CEFE, Université de Montpellier, CNRS, EPHE, IRD, Institut Agro, Montpellier, France; 5 CEFE, Université de Montpellier, CNRS, EPHE, IRD, Montpellier, France; 6 University of Warwick, UK

**Keywords:** Disease, immunity, intraspecific mixture, neighbour, *Oryza sativa*, plant–plant interactions, rice, *Triticum turgidum*, wheat

## Abstract

As part of a trend towards diversifying cultivated areas, varietal mixtures are subject to renewed interest as a means to manage diseases. Besides the epidemiological effects of varietal mixtures on pathogen propagation, little is known about the effect of intraspecific plant–plant interactions and their impact on responses to disease. In this study, genotypes of rice (*Oryza sativa*) or durum wheat (*Triticum turgidum*) were grown with different conspecific neighbours and manually inoculated under conditions preventing pathogen propagation. Disease susceptibility was measured together with the expression of basal immunity genes as part of the response to intra-specific neighbours. The results showed that in many cases for both rice and wheat susceptibility to pathogens and immunity was modified by the presence of intraspecific neighbours. This phenomenon, which we term ‘neighbour-modulated susceptibility’ (NMS), could be caused by the production of below-ground signals and does not require the neighbours to be infected. Our results suggest that the mechanisms responsible for reducing disease in varietal mixtures in the field need to be re-examined.

## Teaser

Susceptibility to fungal pathogens and plant immunity in rice and wheat can be modulated by the presence of intraspecific neighbours and does not require the neighbours to be infected.

## Introduction

Increasing biodiversity in cultivated systems is considered as a promising approach for improving agricultural sustainability (Tilman *et al.*, 2002), in particular to mitigate the impact of diseases (Stenberg, 2017). For instance, mixing varieties of a single species has been successfully applied at the field level to control major foliar pathogens, including *Septoria* disease and leaf rust in wheat (Kristoffersen *et al.*, 2020) and blast fungus in rice (Zhu *et al.*, 2000; Raboin *et al.*, 2012). In such varietal mixtures, well-known resistance genes have strong negative impacts on pathogen dispersal (Burdon *et al.*, 2014), and the resulting resistance is less easily prone to breakdown (Garrett and Mundt, 1999; Burdon *et al.*, 2014). However, choosing varietal components that have high mixing abilities remains a challenge (Barot *et al.*, 2017). Selecting varietal mixtures where immunity is finely regulated by plant–plant interactions (Zhu and Morel, 2019; Pélissier *et al.*, 2021) could provide a means to enhance resistance and its durability. Addressing the very poorly studied question of the physiological effects of varietal mixtures requires experiments under controlled environments that are independent of the processes affecting pathogen propagation that reduce susceptibility at the field scale.

Plants possess a basal immune system that is constitutively expressed at low levels, and can be induced by pathogens (Jones and Dangl, 2006). Genes related to pathogenesis are among those typically induced during this basal immune response (Jones and Dangl, 2006; Ali *et al.*, 2018). The constitutive and inducible expression of this immune system confers basal levels of immunity and leads to reduced susceptibility. Depending on the pathogen lifestyle (biotrophic or necrotrophic), different signalling pathways are involved for triggering basal immunity (De Vleesschauwer *et al.*, 2014). Abiotic factors in the environment in particular can modulate basal immunity (Nobori and Tsuda, 2019). Neighbours such as non-pathogenic microbial organisms surrounding the plant can also affect basal immunity and susceptibility to subsequent pathogen attack, a phenomenon called ‘microbiota-modulated immunity’ (Vannier *et al.*, 2019). However, little attention has been paid to plants themselves as neighbours that can potentially modulate basal immunity and pathogen susceptibility (Stenberg, 2017).

The limited reports that are available indicate that plant–plant interactions can modify immunity and susceptibility to pathogens (Zhu and Morel, 2019; see Pélissier *et al.*, 2021, for a review). Plant–plant interactions can occur between conspecifics (intraspecific interactions) or heterospecifics (interspecific interactions) and can be subdivided into different categories depending on whether they result from direct signalling between plants (chemical or physical signals exchanged) or are mediated by another, third biological agent (e.g. moving pathogen or microbiome), or whether they require that the neighbour is healthy or not (e.g. wounded or infected). On the one hand, indirect interactions involving biological agents moving from sick plants to healthy ones have been documented in several instances in both inter- and intraspecific interactions (Zhu and Morel, 2019; Pélissier *et al.*, 2021). The way these interactions increase resistance is that spores of a pathogen adapted to a host plant migrate to a neighbouring plant to which it is not adapted, thereby stimulating basal immunity and preparing this plant for later infections (‘premunition’). On the other hand, direct interactions are well illustrated by volatile organic compounds that are produced by attacked or wounded plants and are transported to neighbouring, healthy plants that, in turn, develop immunity and resistance (Heil and Karban, 2010; Schuman *et al.*, 2015; Ninkovic *et al.*, 2019). This has been shown in both inter-and intra-specific plant–plant interactions, in particular against insects (Karban *et al.*, 2000, 2006). In the case of intraspecific interactions, such a phenomenon is called ‘eavesdropping’ (Rebolleda-Gómez and Wood, 2019) where a previously unaffected plant primes its defenses after perceiving a signal emitted by the neighbouring, attacked plant. While there are many report that sick or attacked plants can signal danger to neighbours, thus triggering their immunity and reducing their susceptibility (Cheol Song *et al.*, 2016; Wenig *et al.*, 2019), very few studies have indicated that heathy plants can directly affect immunity in their plant neighbourhood.

Plant–plant interactions are also mediated by alterations of resource availability. For instance, the reduction of light availability provoked by the shade of neighbours can alter immunity and susceptibility to pathogens (‘shade-avoidance syndrome’; Ballaré and Pierik, 2017). The plant density of the neighbourhood has also been shown to affect immunity (e.g. Chen *et al.*, 2019). In addition to density and the associated shade-avoidance syndrome, the identity of the neighbour has been shown to affect immunity (Subrahmaniam *et al.*, 2018; Zhu and Morel, 2019). For instance, Arabidopsis grown in the presence of healthy *Hieracium pilosela* shows modifications to its transcriptome that are similar to the those observed during pathogen infection (Schmid *et al.*, 2013). It has also been reported that cultivation of maize and pepper together increases the expression of defense-related genes in maize and its resistance to pathogens (Ding *et al.*, 2015). Thus, simply the presence of healthy plants can affect basal immunity and resistance to pathogens in neighbouring plants of another species. In the case of intraspecific interactions, it was shown that root exudates from Arabidopsis can modify the expression of defense-related genes in conspecific, genetically distinct plants (Biedrzycki *et al.*, 2011). Apart from this indirect evidence that intraspecific interactions modulate immunity, there is, to our knowledge, no direct evidence that signals emitted by healthy plants can modulate the susceptibility to pathogens or immunity in conspecific, genetically distinct neighbours. Establishing whether some conspecific neighbours rather than others enhance immunity and susceptibility to pathogens will clearly have important implications for designing varietal mixtures. Here, we designed a pioneering study aimed at determining whether intraspecific interactions with diseased or healthy neighbours can affect basal immunity and susceptibility to pathogens. We chose individual genotypes of rice and durum wheat as subjects to conduct greenhouse experiments in which plants were grown with various genetically different, conspecific neighbours. After identifying neighbours that affected susceptiblity in the plants in question, we measured their global impact on disease susceptibility and their capacity to modulate the expression of basal immunity genes, and evaluated the type of signal underlying the observed modifications.

## Materials and methods

### Plant material, growth conditions, and experimental design

An overview of the experimental design is shown in Supplementary Fig. S1 and Supplementary Table S1.

We used the temperate rice (*Oryza sativa* subsp. *japonica*) genotype Kitaake (KIT) and the durum wheat (*Triticum turgidum*) genotype Cultur (CUL) as the subjects on which we measured phenotypic responses. These are termed the ‘focal’ genotypes whilst other genotypes are termed ‘neighbours’. In an initial trial, we examined the interactions of the two focal genotypes with nine others of the same species (Supplementary Tables S2, S3). All the genotypes had comparable levels of susceptibility to the pathogens that we used (Supplementary S1C; see below). KIT and CUL were grown in the presence of plants either of the same genotype (termed ‘pure’ conditions) or of a different genotype (mixture), giving 10 combinations in each species (1 pure + 9 mixture). As a result of this initial trial, we selected the rice cultivar Lido (LID) and the durum wheat cultivar Atoudur (ATO) as representative neighbours for further detailed examination.

For rice, plants were grown in plastic pots with one row of four focal plants and one row of four neighbours. The pots were 9×9×9.5 cm and filled with a substrate of 58% blond peat, 18% coconut powder, 10% perlite, 9% volcanic sand, and 5% clay, supplemented with 3.5 g l^–1^ of fertilizer (Basacote Native 6M, NPK 14-3-19). The plants were grown under a 16/8-h photoperiod with artificial light of 55 000 lumen at 27/23 °C. For wheat, plants were grown in plastic pots with one row of three focal plants and one row of three neighbours. The pots were 7×7×6 cm and filled with a substrate of 80% of N2 Neuhaus soil (ID 4020.20) and 20% pozzolan supplemented with 4.5 g l^–1^ fertilizer (Flocoat Polyon, NPK 17-7-27, Florenprodi, Paris). The plants were grown under a 16/8-h photoperiod with artificial light of 35 000 Lumen), at 24/20 °C. The equivalent population densities of ~1 plant per 10 cm^–2^, which were about half those typically used in experiments (e.g. Berruyer *et al.*, 2003), and the high quantities of nutrients provided resulted in low levels of competition.

To test the relative impacts of inter- and intra-genotypic interactions, we also established pots in the same system of cultivation in which a single focal plant and a single neighbour of the same genotype were planted.

### Soil sterilization, and below- and above-ground separation treatments

To examine the effects of the soil microbiota on neighbour-modulated susceptibility, we sterilized the soil by autoclaving the substrate for 1 h at 120°C before sowing and the addition of fertilizer. The results were compared with those of all the other experiments where non-autoclaved soil was used. 

We examined the effects of completely separating the roots of the plants by placing a non-porous plastic membrane between the focal and neighbour plants. We also examined the effects of separating the roots of the focal and neighbour plants with a porous membrane (22–25 μm; Merck Miilipore) that prevented physical contact but allowed movement of water and chemicals.

We also performed experiments to separate the above-ground parts of the plants by placing paper bags over the neighbours (Supplementary Fig. S1A). There were two diseased-neighbour treatments of ‘Neighbour inoculated’ and ‘Neighbour inoculated and covered’, compared with a control where the neighbours were covered only whilst the inoculation procedure was being carried out to avoid them being infected (‘Neighbour non-inoculated’).

### Pathogen material, inoculation procedure, and disease assessment

For rice, we selected the multivirulent strain CL26 (Gallet *et al.*, 2015) of the hemibiotrophic fungal pathogen *Magnaporthe oryzae*. The strain was grown for 10 d on rice flour agar medium (20 g of rice flour, 15 g of agar, 2.5 g of yeast extract, and 1 l of distilled water) under fluorescent light (alternating 3250 lumen white and 1400 purple neon lights) with a 12-h photoperiod at 26 °C. We harvested conidia by flooding the culture plates with 5 ml of sterile distilled water and filtering with a Miracloth 22–25 μm filter. Plants were inoculated at 3 weeks old. Trays containing 15 pots (i.e. 120 plants) were sprayed with 30 ml of a suspension of 100 000 conidia ml^–1^ (with 0.1% gelatin) as described by Berruyer *et al.* (2003), representing a rate of ~ 25000 conidia per plant.

We used the hemibiotrophic bacterial strain PXO99A of *Xanthomonas oryzae* pv. *oryzae* to inoculate all the individual rice plants by leaf clipping. The bacteria were cultivated for 2 d at 28 °C in the dark, in PSA medium (10 g peptone, 10 g sucrose, 1 g glutamic acid, 16 g agar, 1l H_2_O). We performed leaf-clip inoculation on 4- to 5-week-old plants using a bacterial suspension with an OD_600_ of 0.2 and measured the size of lesions at 15 d post-inoculation (dpi) as described by Oliva *et al.* (2019).

We also inoculated rice with the necrotrophic fungus *Bipolaris oryzae* (De Vleesschauwer *et al.*, 2014), using the strain FR9037 isolated on rice in Camargue in the south of France. The strain was grown for 14 d on RFA (Rabbit Food Agar) medium (30 g l^–1^ agar, 100 g l^–1^ Forti Diet Pro Health, Kaytee) under the same culture conditions that are described above for *M. oryzae*, with a 12-h photoperiod at 25 °C (Hau and Rush, 1980). The conidia were harvested and filtration and inoculation were performed using the same protocol as for *M. oryzae*, at a rate of ~15000 conidia per plant.

We inoculated durum wheat plants with the causal agent of leaf rust, the biotrophic fungus *Puccinia* triticina (Bolton *et al.*, 2008), using a multivirulent field isolate from southern France. Because *P. triticina* is an obligatory biotroph, we harvested spores by aspiration from infected plants in a separate greenhouse and immediately froze them at –80 °C. To prepare the inoculum, a heat shock of 40 °C for 10 min was applied to the spores and they then were suspended in 1 ml of Tween 20 before diluting in 30 ml of water (with 0.1% gelatin). We used an average of 8 mg of spores to inoculate 144 plants by spraying (Ballini *et al.*, 2020).

We also inoculated durum wheat with the P1 isolate of the hemibiotrophic fungus *Zymoseptoria tritici* (synonym *Septoria tritici*) obtained from a durum wheat line Pescadou in 2015 in Montpellier, France. We cultivated the strain over 4–5 d under the same conditions that are described above for *M. oryzae*, except that the temperature was 20 °C, on YPD medium (yeast extract 10 g l^–1^, bactopeptone 20 g l^–1^, sucrose 20 g l^–1^, agar 15 g l^–1^). Spores were havested by flooding the culture plate with 5 ml of sterile distilled water, and one drop of Tween 20 was add for 10 ml of suspension. We performed inoculation with a solution of 10^6^ spores ml^−1^ that was applied to the leaves of all the individual plants using a paintbrush (Kettles and Kanyuka, 2016).

In all cases, an inoculation solution (0.1% gelatin) without spores was used as a control.

After inoculation, the rice and wheat plants were incubated for 16 h in the dark in a controlled-climate chamber at 25 °C and 95% relative humidity, and then returned to normal growth conditions. At 6–7 d after inoculation (21 d for *Septoria* disease in wheat), we scanned the youngest fully-emerged leaf of 3–4 focal plants per pot (600 pixels per inch; Epson Perfection V370 Photo scanner). Abnormal plants were not scored. The images were analysed using the R package LeAFtool (Lesion Area Finding tool; https://github.com/sravel/LeAFtool), which measures leaf area and lesion number. The parameters used for the analysis were at least 10 000 pixels for leaves and 50 pixels for lesion areas, with the blur value at 1. To account for outliers and software errors, we manually removed from the analysis lesions that we considered to be of abnormal size. Leaf susceptibility was then estimated as the number of lesions cm^–2^ of leaf area (or percentage of leaf necrosis area for *X. oryzae* in rice and *Z. tritici* in wheat).

### RNA extraction and RT-qPCR analysis

For gene expression studies, we used protocols described previously by Delteil *et al.* (2012). Frozen leaf tissues were ground in liquid nitrogen and ~500 mgof powder was treated with 1 ml of TRIzol (Invitrogen). RNA samples (5 μg) were denatured for 5 min at 65 °C with oligo(dT) 18 (3.5 mM) and deoxynucleoside triphosphate (dNTP; 1.5 mM). They were then subjected to reverse transcription for 60 min at 37 °C with 200 U of reverse transcriptase M-MLV (Promega) in the appropriate buffer. The cDNA (5 μg, dilution 1:10) was then used for reverse-transcription quantitative (RT-q)PCR. The RT-qPCR mixtures contained PCR buffer, dNTP (0.25 mM), MgCl2 (2.5 mM), forward and reverse primers (final concentrations of 150, 300, or 600 nM), 1 U of HotGoldStar polymerase, and SYBR Green PCR mix according to the manufacturer’s recommendations (Eurogentec, Seraing, Belgium). The genes examined and the primers used are given in Supplementary Tables S3, S4. Amplification was performed as follows: 95 °C for 10 min; 40 cycles of 95 °C for 15 s, 62 °C for 1 min, and 72 °C for 30 s; 95 °C for 1 min and 55 °C for 30 s. RT-qPCR was performed using a LightCycler480 instrument (Roche) and data were extracted using the accompanying software. The rice *actin* gene (*Os03g50890*) for and durum wheat *ubiquitin* gene (*CD921597*) were used as internal controls, and their expression levels did not vary significantly between treatments (Supplementary Fig. S2). The calculation of gene expression was performed using the measured efficiency for each primer pair as described by Vergne *et al*. (2007). The defense genes that we examined are proxies for basal immunity in rice (Vergne *et al.*, 2010; Peng *et al.*, 2017) and wheat (Duba *et al.*, 2018). The expression of genes was measured before inoculation and at different time-points afterwards depending on the pathogen infection cycle.

### Statistical analyses

All analyses were performed using R (www.r-project.org). All experiments were repeated at least three times, and each experiment included at least four replicate pots for each focal/neighbour association, representing a total of 2964 plants for rice and 2340 for wheat. We used a linear model where the number of lesions per unit area of leaf in the focal plant was a function of the experiment effect, the position effect (placement of the pot in the experiment), and the genotypic identity of the neighbouring plant. This model was used to calculate least-square means (LSmeans) using the R package *lsmeans*. Square-root transformation was used to correct for normality and homocedasticity. The impact of the neighbour on a focal plant was tested by ANOVA followed by Dunnett’s test (function *glht* in the R package *multcomp*). The ‘pure’ condition was used as the control group for Dunnett’s tests. For gene expression, at least one of the distributions was not normal, and therefore we evaluated differences between treatments using non-parametric Wilcoxon’s tests.

## Results

### Varietal mixtures affect disease susceptibility in the absence of pathogen propagation

**Fig. 1.**
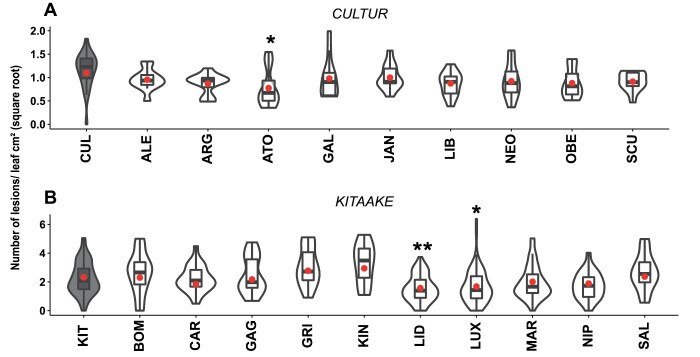
Disease susceptibility of durum wheat and rice in intraspecific mixtures. (A) Plants of the wheat genotype Cultur (CUL) and (B) the temperate *japonica* rice genotype Kitaake (KIT) were grown with neighbours of the same genotype (grey shading, ‘pure’ condition) or of a conspecific genotype (white shading, mixture), and the plants were inoculated with *Puccinia triticina* (leaf rust disease) for wheat and *Magnaporthe oryzae* (blast disease) for rice (see Methods). For genotype abbreviations see Supplementary Tables S2 and S3. Susceptibility was measured as the number of lesions cm^–2^ of leaf area on the CUL and rice KIT focal plants (data are square-root transformed). The violin plots represent at least *n*=42 plants for rice and *n*=36 plants for wheat. The red dots represent the least-square means as determined using a linear model. For wheat, each combination was performed eight times in three separate experiments, and for rice, each combination was performed 12 times in three separate experiments. Significant differences compared with the ‘pure’ control were determined using ANOVA of the linear model followed by Dunnett’s tests: *P<0.05; **P<0.01.

We first evaluated the disease susceptibility of one focal plant genotype of each species in the presence of nine different conspecific neighbours. In durum wheat, the susceptibility to *P. triticina* of the focal genotype, Cultur (CUL), was significantly reduced by 30% in the presence of the neighbour genotype Atoudur (ATO) comapred with the control (CUL/CUL; Fig. 1A). In rice, the susceptibility to *M. oryzae* of the focal genotype, Kitaake (KIT) was significantly reduced by 32% in the presence of the neighbour genotype, Lido (LID) and by 27% in the presence of Luxor (LUX) compared with the KIT/KIT control (Fig. 1B). We therefore selected the wheat CUL/ATO and rice KIT/LID focal/neighbour mixtures as models for further experiments.

In addition to inter-genotypic interactions between focal and neighbour plants, intra-genotypic interactions within focal plants could also occur in our experimental system. We tested this possibility by comparing susceptibility levels in our standard design (three focal plants plus three neighbour plants for wheat, four plus four plants for rice) with the situation where only inter-genotypic interactions could occur (i.e. one focal plant with one neighbour plant only). We observed no differences in the response of susceptibility triggered by the neighbour between the two designsdesigns (Supplementary Fig. S3), suggesting that the reduction of susceptibility that we observed in our standard design (Fig. 1) was only due to inter-genotypic interactions.

### Varietal mixtures have a broad impact on disease susceptibility

**Fig. 2.**
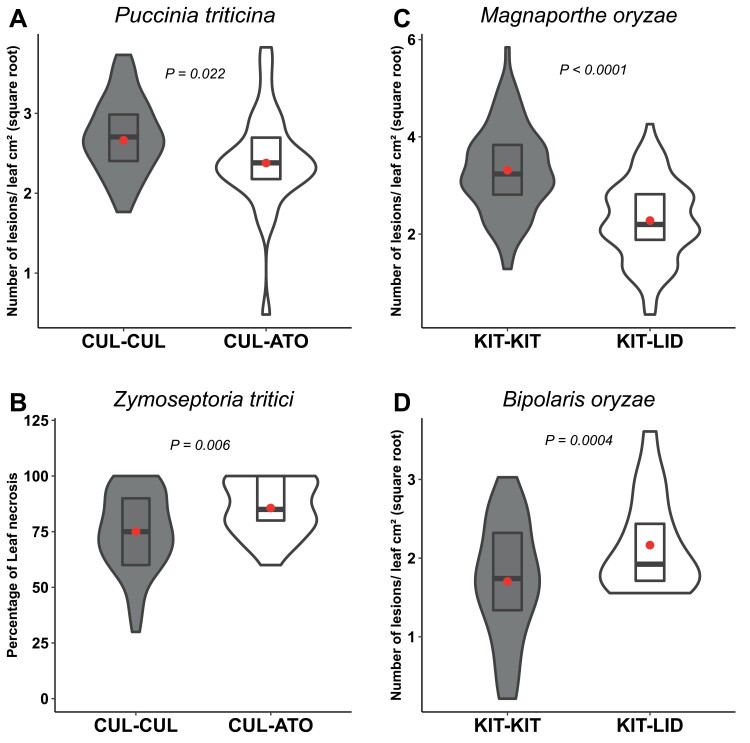
Impact of conspecific mixtures of durum wheat and rice genotypes on disease susceptibility to different fungal pathogens. (A, C) Plants of the wheat genotype Cultur were grown either with itself (CUL-CUL, ‘pure’ condition) or with the genotype Atoudur (CUL-ATO), and (B, D) plants of the temperate *japonica* rice genotype Kitaake were grown either with itself (KIT-KIT, ‘pure’) or with the genotype Lido (KIT-LID). The plants were inoculated with the fungal pathogens as indicated (see Methods). *Puccinia triticina* is a biotrophic fungus whlist *Zymoseptoria tritici*, *Magnaporthe oryzae*, and *Biopolris oryzae* are all hemibiotrophic. All measurements were made on the CUL and KIT focal plants. Susceptibility was measured as the number of lesions cm^–2^ of leaf area (data are square-root transformed), except for *Z. tritici* were it was measured as percentage of leaf necrosis. The violin plots represent at least *n*=42 plants for rice and *n*=36 plants for wheat. The red dots represent the least-square means as determined using a linear model. The data represent at least three experiments of four and six replicates for wheat and rice, respectively. Significant differences were determined using ANOVA followed by Dunnett’s tests of the linear model. The corresponding data with ATO and LID as the focal plants are given in Supplementary Fig. S5.

We tested the impact of the model mixtures CUL/ATO and KIT/LID on susceptiblity to different pathogens displaying contrasting lifestyles. As expected, compared with the controls, both model mixtures displayed reductions of susceptibility in focal plants when inoculated with the pathogens that were used in the initial test: in wheat susceptibility to the biotrophic *P. triticina* was reduced by 11% in CUL (Fig. 2A) while in rice susceptibility to the hemibiotrophic *M. oryzae* was reduced by 34% in KIT (Fig. 2C). In contrast, wheat susceptibility to the hemibiotrophic fungal pathogen *Z. tritici* was increased by 14% in the mixture compared to the pure condition (Fig. 2B). For the rice KIT/LID mixture, we tested the hemibiotrophic bacterial pathogen *X. oryzae* and the necrotrophic fungal pathogen *B. oryzae*. While susceptiblity to *X. oyzae* was not affected in KIT (Supplementary Fig. S4A), the susceptibility to *B. oryzae* increased by 46% when KIT plants were grown in the presence of LID conspecific plants (Fig. 2D).

In our experimental system, susceptibility could be evaluated for each member of the pair of genotypes. We therefore examined whether the observed changes in pathogen susceptibility in the CUL and KIT focal plants were associated with changes in susceptibility in their respective ATO and LID neighbour plants. In wheat, both components of the pair showed reduced susceptibility to *P. triticina* (Fig. 2A, Supplementary Fig. S5A) and increased susceptibility to *Z. tritici* (Fig. 2B, Supplementary Fig. S5B). In rice, both members of the pair showed reduced susceptibility to *M. oryzae* (Fig. 2C, Supplementary Fig. S5C) and increased susceptibility to *B. oryzae* (Fig. 2D, Supplementary Fig. S5D). The susceptibility of rice to the bacterial pathogen *X. oryzae* was marginally reduced in LID in the pair KIT/LID (Supplementary Fig. S4B). Thus, both members of the pairs were generally affected by being in mixtures, and opposite effects on disease susceptibility could occur.

### Requirements for triggering changes in susceptibility in varietal mixtures

The plant–plant interactions behind the changes in susceptibility that we observed in the wheat CUL/ATO and rice KIT/LID model mixtures could have taken place above and/or below ground. In addition, it was also possible that inoculation of the neighbour plants could have been required to trigger the changes in susceptibility of the focal plants. To address these questions, we set up experiments where we limited or abolished physical contact between the roots, the presence of soil microbiota, or the transfer of a signal by the neighbours. We examined inoculation with *P. triticina* in wheat and with *M. oryzae* in rice.

**Fig. 3.**
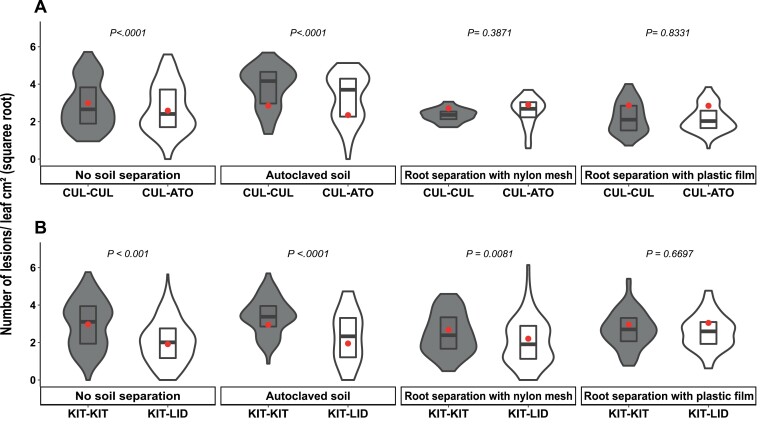
Effects of soil sterilization and root separation on disease susceptibility of durum wheat and rice genotypes grown in different conspecific mixtures. (A) Plants of the wheat genotype Cultur were grown either with itself (CUL-CUL, ‘pure’ condition) or with the genotype Atoudur (CUL-ATO) and were inoculated with *Puccinia triticina*. (B) Plants of the temperate *japonica* rice genotype Kitaake were grown either with itself (KIT-KIT, ‘pure’) or with the genotype Lido (KIT-LID) and inoculated with *Magnaporthe oryzae*. Susceptibility was measured as the number of lesions cm^–2^ of leaf area on the CUL and rice KIT focal plants (data are square-root transformed). The plants were grown in normal soil without any root separation, in autoclaved soil without any root separation, in normal soil with roots separated by a porous nylon mesh, and in normal soil with roots separated by a non-porous plastic film (see Methods). The violin plots represent at least *n*=42 plants for rice and *n*=36 plants for wheat. The red dots represent the least-square means as determined using a linear model. For wheat, each combination was performed eight times in three separate experiments, and for rice, each combination was performed 12 times in three separate experiments. Significant differences were determined using ANOVA of the linear model followed by Dunnett’s tests.

The reduction of susceptibility induced by neighbours in the rice and wheat mixtures remained significant when the soil had been autoclaved prior to sowing (Fig. 3). In contrast, placing a non-porus plastic membrane in the soil between the focal and neighbour plants removed the changes in disease susceptibility in both the wheat and rice mixtures. Finally, placing a porous membrane between the plants did not modify the changes in susceptibility in the the KIT/LID mixture (Fig. 3B) whereas it removed them in the CUL/ATO mixture (Fig. 3A).

**Fig. 4.**
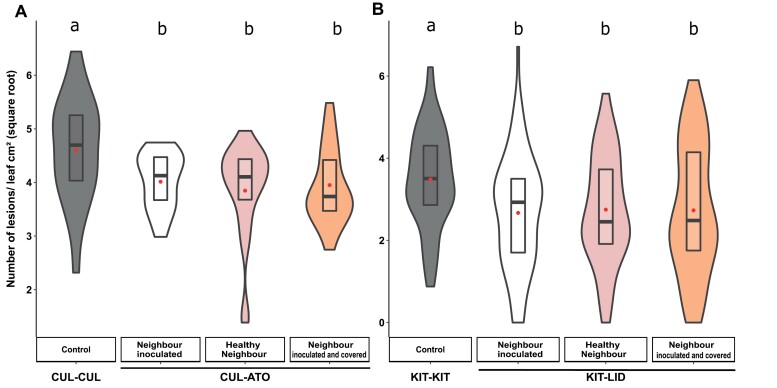
Effects of healthy and diseased neighbours on disease susceptibility of durum wheat and rice genotypes grown in different conspecific mixtures. (A) Plants of the wheat genotype Cultur were grown either with itself (CUL-CUL, ‘pure’ condition) or with the genotype Atoudur (CUL-ATO) and were inoculated with *Puccinia triticina*. (B) Plants of the temperate *japonica* rice genotype Kitaake were grown either with itself (KIT-KIT, ‘pure’) or with the genotype Lido (KIT-LID) and inoculated with *Magnaporthe oryzae*. Susceptibility was measured as the number of lesions cm^–2^ of leaf area on the CUL and rice KIT focal plants (data are square-root transformed). In each case, plants were also grown with inoculated neighbours that were covered to limit aerial contact (see Methods). The violin plots represent at least *n*=42 plants for rice and *n*=36 plants for wheat. The red dots represent the least-square means as determined using a linear model. For wheat, each combination was performed eight times in three separate experiments, and for rice, each combination was performed 12 times in three separate experiments. Different letters indicate significant differences as determined using ANOVA followed by Tukey’s HSD tests of the linear model (*P*<0.05).

The changes in susceptibility in the focal plants were not modified when infection of the neighbour plants was prevented by covering them during inoculation (Fig. 4), and no differences were observed between having healthy and inoculated neighbours. In addition, there was no effect when a cover was placed over the neighbours after inoculation.

### Varietal mixtures affect the expression of basal immunity

**Fig. 5.**
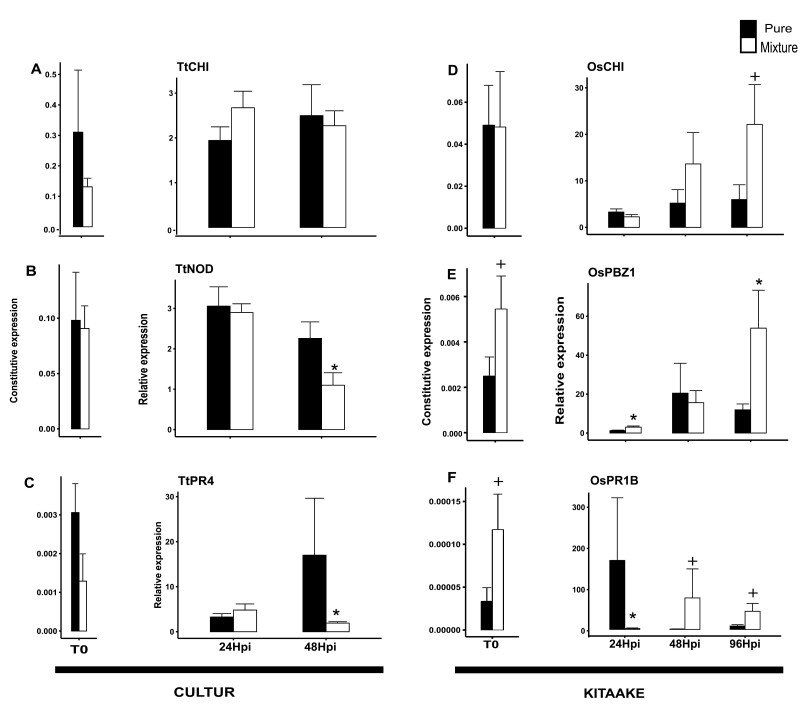
Expression of immunity-related genes in response to fungal pathogens in durum wheat and rice genotypes grown in different conspecific mixtures. (A–C) Plants of the wheat genotype Cultur were grown either with itself ( ‘pure’ condition) or with the genotype Atoudur (mixture) and were inoculated with *Puccinia triticina*. (D–F) Plants of the temperate *japonica* rice genotype Kitaake were grown either with itself (‘pure’) or with the genotype Lido (mixture) and inoculated with *Magnaporthe oryzae*. The ‘pure’ condition and the mixtures are as indicated in the key. Tt, durum wheat (*Triticum turgidum*); Os, rice (*Oryza sativa*). Gene expression was measured in leaves before infection (T0) and at 24 h post-inoculation (hpi) and 48 hpi for wheat, and at 24, 48, and 96 hpi for rice. Expression was determined by RT-qPCR and normalized using the *actin* and *ubiquitin* genes for rice and wheat, respectively. The constitutive expression is shown for T0, and for the subsequent time-points expression is relative to that at T0 (i.e. ratio of inoculated/non-inoculated). Data are means (SE) of at least *n*=6 replicates. Significant differences between the ‘pure’ conditions and the mixtures were determined using Wilcoxon tests: ^+^*P*<0.1; *P<0.05. The corresponding data with Atoudur and Lido as the focal plants are given in Supplementary Fig. S6.

We examined whether the changes in susceptibility in the CUL/ATO and KIT/LID mixtures were associated with changes in the expression of basal immunity genes before or after inoculation. The expression data for CUL and KIT as focal plants are shown in Fig. 5 and the reciprocal data for ATO and LID as focal plants are provided in Supplementary Fig. S6.

For the wheat CUL and ATO genotypes, no significant differences in the expression of defense genes were found between the pure and mixture conditions before inoculation. After inoculation, *TtNOD* and *TtPR4* were significantly less induced at 48 h in CUL focal plants in the mixture than in CUL plants grown in the pure condition. Interestingly, the opposite pattern was found at 48 h after infection in the ATO plants in the ATO/CUL mixture.

For the rice KIT/LID mixture before inoculation, *OsPBZ1* and *OsPR1B* had marginally higher expression (*P*<0.1) in the KIT plants, and *OsPBZ1* had significantly higher expression in the LID plants. After inoculation, a significant increase in expression was observed in the KIT plants for *OsPBZ1* at both 24 h and 96 h after inoculation and for *OsPR1B* at 96 h in the KIT/LID mixture for KIT plants. No significant differences were found for the LID plants.

## Discussion

Our results show direct evidence that, for the two crop species examined, varietal mixtures can constitutively modify plant susceptibility to various foliar pathogens, both intrinsically and independently of pathogen propagation, and as a consequence of inter-genotypic, conspecific plant–plant interactions. The major phenotypical changes that we observed were accompanied by some transcriptional changes in genes related to the immune response, although these were limited. Moreover, we show that a signal from healthy neighbours is involved, probably below-ground, suggesting that the shade avoidance syndrome is not involved here. To date, constitutive plant–plant interactions have been shown to affect disease susceptibility in interspecific mixes (Zhu and Morel, 2019; Pélissier *et al.*, 2021), and our experiments provides the groundwork for studying this phenomenon in intraspecific mixes. We propose that these phenomenoa in response to pathogens be termed plant ‘neighbour-modulated susceptibility’ (NMS), by analogy with microbiota-modulated immunity (MMI; (Vannier *et al.*, 2019)). Here, we found that intraspecific NMS was present in 10–20% of the cases examined (Fig. 1), demonstrating that the genetic identity of the neighbours matters. This opens the possibility for conducting genetic analyses of intraspecific NMS, a promising approach for discovering the genes underlying these plant–plant interactions. For instance, testing the effect of a wide range of genotyped neigbours on one given focal plant could lead to identification of the genes involved in the triggering of NMS.

There are numerous reports of changes in susceptibility to insects following plant–plant interactions in intraspecific mixtures (e.g. Heil and Karban, 2010), but data remain scarce for pathogen susceptibility. Furthermore, most previous studies have required prior inoculation of the neighbours to induce changes in the focal plant. For instance, it has recently been shown that Arabidopsis plants infected with a bacterial pathogen produce an aerial signal that is translocated onto uninfected neighbours and primes their defenses, leading to reduced susceptibility (Wenig *et al.*, 2019). In our experimental system, there was no difference in NMS for plants with either healthy or inoculated neighbours (Fig. 4). This demonstrates that changes in susceptibility in intraspecific plant mixtures does not require inoculation of the neighbour, and thus does not depend on the neighbour’s susceptiblity level, and hence expression of immunity can be constitutive. In that respect, NMS does not resemble the many cases of ‘eavesdropping’ situations reported so far (Rebolleda-Gómez and Wood, 2019).

While several studies have examined changes in expression of genes related to immunity within interspecific plant–plant interactions (Zhu and Morel, 2019; Pélissier *et al.*, 2021), data are currently scarce and indirect for intraspecific interactions (Biedrzycki *et al.*, 2011). Our results showed that intraspecific plant–plant interactions can modify the expression of defense genes, both before and after pathogen attack. This was particularly evident in the rice KIT genotype in the KIT/LID mixture (Fig. 5D–F). The expression of *OsCHI* was not affected in a constitutive manner but only after infection, while expression of *OsPR1b* and *OsPBZ1* was increased by 2–3-fold before infection and by 3–30-fold after infection. This suggests that defense had been primed (Martinez-Medina *et al.*, 2016) by the presence of neighbours and not just constitutively enhanced. The hypothesis can be made that the effects of the plant–plant interactions on susceptibility resulted from some of the observed changes in gene expression. For instance, in rice the LID neighbour induced the expression of the *OsPBZ1* and *OsPR1B* defense genes in the KIT focal plants. The expression of these two genes has been shown to correlate with the hypersensitive response (Takahashi *et al.*, 1999), which is known to promote the development of necrotrophic pathogens (van Kan, 2006; Mengiste, 2012) and to reduce the development of hemibiotrophic ones (Jia *et al.*, 2000; Fan *et al.*, 2018). This is consistent with the increased susceptibility to *B. oryzae* and the reduced susceptibility to *M. oryzae* that we observed in rice mixtures (Fig. 2C, D). More generally, the fact that pathogens with different lifestyles were affected in opposite ways by the plant–plant interactions in our study indicates a role of immunity in intraspecific NMS. Indeed, several defense pathways controlled by different defense hormones can have antagonistic effects on infection depending on the lifestyle of the pathogen (De Vleesschauwer *et al.*, 2014; Ding *et al.*, 2016). However, the fact that the susceptibility of rice genotypes to *X. oryzae* was affected in an opposite way to *M. oryzae* (Fig. 2C, D) and that the expression of durum wheat defense genes was only weakly affected, and even reduced when plants were grown in mixtures (Fig. 5A–C, Supplementary Fig. S6A–C), suggests that immunity was not the only driver of NMS. These observations highlight the need for further investigation of the molecular physiology of varietal mixtures. Measurements of defense hormones and more exhaustive transcriptomic analyses are required in order to understand the overall impact of intraspecific plant–plant interactions on plant physiology.

Modifications of the susceptibility responses to pathogens driven by plant–plant interactions can be caused by above- and/or below-ground signalling processes (Zhu and Morel, 2019; Pélissier *et al.*, 2021). The experiments that we designed to examine the requirements for intraspecific NMS showed that the interactions were located in the soil (Fig. 3), although with slightly different responses between rice and wheat. Indeed, NMS required direct root contact in the case of wheat but not in the case of rice mixtures. NMS did not require the presence of microbiota in the soil for either species (Fig. 3), although we cannot exclude the possible effects of microbiota transferred to the sterilized soil by the seeds or through the air during the experiments.

The mechanisms that trigger intraspecific NMS in focal plants are still unknown. In mixtures of tree species, plant competition induces a reduction of aerial biomass and an increase in defenses, such as phenolic or terpenoid contents (Donaldson *et al.*, 2006; Fernandez *et al.*, 2016). Thus, plant–plant interactions could indirectly affect susceptibility to pathogens because of a putative trade-off between growth (subsequent to competition) and defense (Huot *et al.*, 2014; Karasov *et al.*, 2017). Chemical signals exchanged in the soil could also be involved in triggering NMS, and have been identified as being the cause of modifications in susceptibility in interspecific plant–plant interactions (Subrahmaniam *et al.*, 2018; Zhu and Morel, 2019; Pélissier *et al.*, 2021). However, there is only indirect evidence to suggest that molecules secreted in root exudates modify plant immunity in intraspecific mixtures (Biedrzycki *et al.*, 2011). One such chemical signal in rice could be allantoin, which is produced by roots and is involved in kin recognition (Yang *et al.*, 2018). Moreover, its production and excretion into the soil can vary depending on the rice genotype (Wang *et al.*, 2007). In wheat, the response to plant competition is commonly measured by the amount of production of DIMBOA, a secondary metabolite (Kong *et al.*, 2018), and since its by-products can activate genes related to stress and defense in Arabidopsis (Venturelli *et al.*, 2015), it represents a good candidate for future studies. Electrical signals have also been shown to be involved in plant root interactions (Volkov *et al.*, 2019) and hence might be worthy of investigation.

Varietal mixtures, resulting from mixing several cultivars of the same species, can reduce disease in crop fields (Mundt, 2002). To date, the positive effects observed in the field have been attributed to epidemiological mechanisms that mostly result from the increase in functional diversity (Garrett and Mundt, 1999; Gaba *et al.*, 2015). Our discovery of neighbour-modulated susceptibility (NMS) suggests that disease reduction in intraspecific mixtures can also result from direct plant–plant interactions, which probably act in parallel with and independently of the other field-scale mechanisms that occur. NMS could provide a new approach to deciding how varieties are mixed and hence how diseases can be managed in agriculture. However, many questions regarding NMS renain to be answered, including whether it lasts throughout the lifespan of the plant, what (if any) is its quantitative contribution to limiting diseases in the field, and can it be manipulated to avoid adverse effects whilst utilizing its positive effects on disease resistance?

## Supplementary data

The following supplementary data are available at *JXB* online.

Fig. S1. Summary of experimental design and examples of leaf symptoms observed.

Fig. S2 Expression of housekeeping genes before and after inoculation of plants.

Fig. S3. Effects of intra- and inter-genotypic plant–plant interactions on disease susceptibility in rice and wheat.

Fig. S4. Responses of selected intra-specific mixtures of rice on disease susceptibility to *Xanthomonas oryzae*.

Fig. S5. Effects of different pathogen lifestyles on responses of disease susceptibility in selected intra-specific mixtures in rice and wheat.

Fig. S6. Expression of immunity-related genes in the wheat ATO genotype and the rice LID genotype when they were the focal plants (reciprocal data for Fig. 5).

Table S1. Summary of the experimental treatments and the outcomes observed.

Table S2. List of durum wheat genotypes used in the study.

Table S3. List of the rice genotypes used in the study.

Table S4. List of durum wheat genes examined in this study.

Table S5. List of rice genes examined in this study.

erab277_suppl_Supplementary_Data

## Data Availability

The data supporting the findings of this study are available from the corresponding author, Jean-Benoit Morel, upon request.
