# Supplementary material for: Plant neighbour-modulated susceptibility to pathogens in intraspecific mixtures
Source: J Exp Bot. 2021 Jun 14;72(18):6570–80. doi: 10.1093/jxb/erab277 (PMC8483782; doi:10.1093/jxb/erab277)
Supplement: erab277_suppl_Supplementary_Data [file erab277_suppl_supplementary_data.pdf]

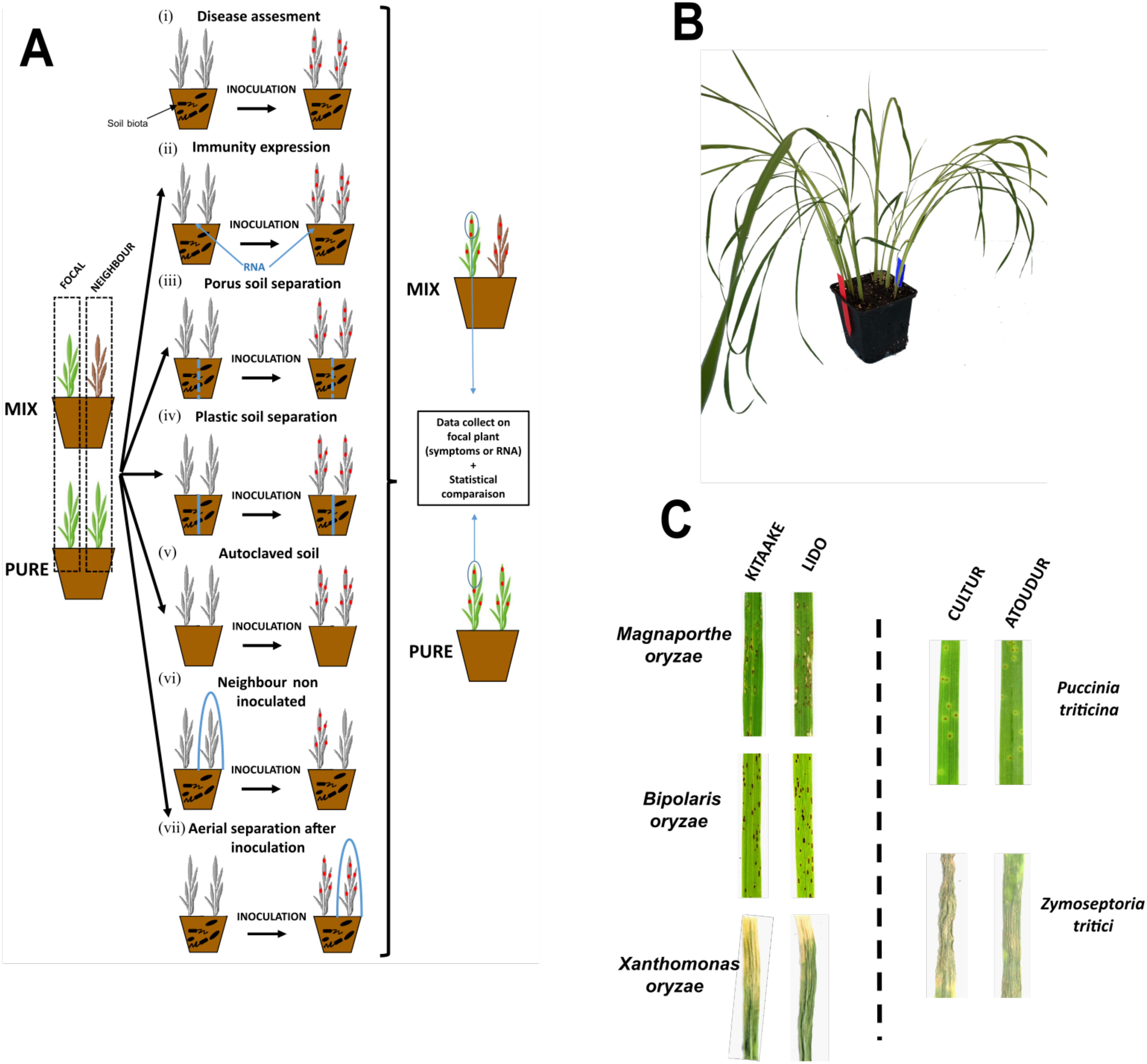

Fig. S1 Overall experimental design used in this study.

(A). Two model mixtures (KITAAKE/LIDO for rice and CULTUR/ATOUDUR for wheat) were selected. Each mixture and pure condition was used for several measurements and treatments: (i) Disease assessment with various pathogens, (ii) RNA harvesting for analysis of defense gene expression before and after inoculation, (iii) porus soil separation, (iv) plastic soil separation, (v) autoclaved soil, (vi) non inoculation of the neighour and (vii) aerial separation after inoculation between focal and neighbour. For all the experiments, symptoms (e.g number of lesions) or RNA were analyzed on the last developed leaf of the focal plant. Results of the focal pla nt in pure and mix condition were compared with the appropriate statistics.

(B). Example of set-up with two rice lines of plant used in one pot.

(C) Examples of leaf symptoms quantified by the pathogens

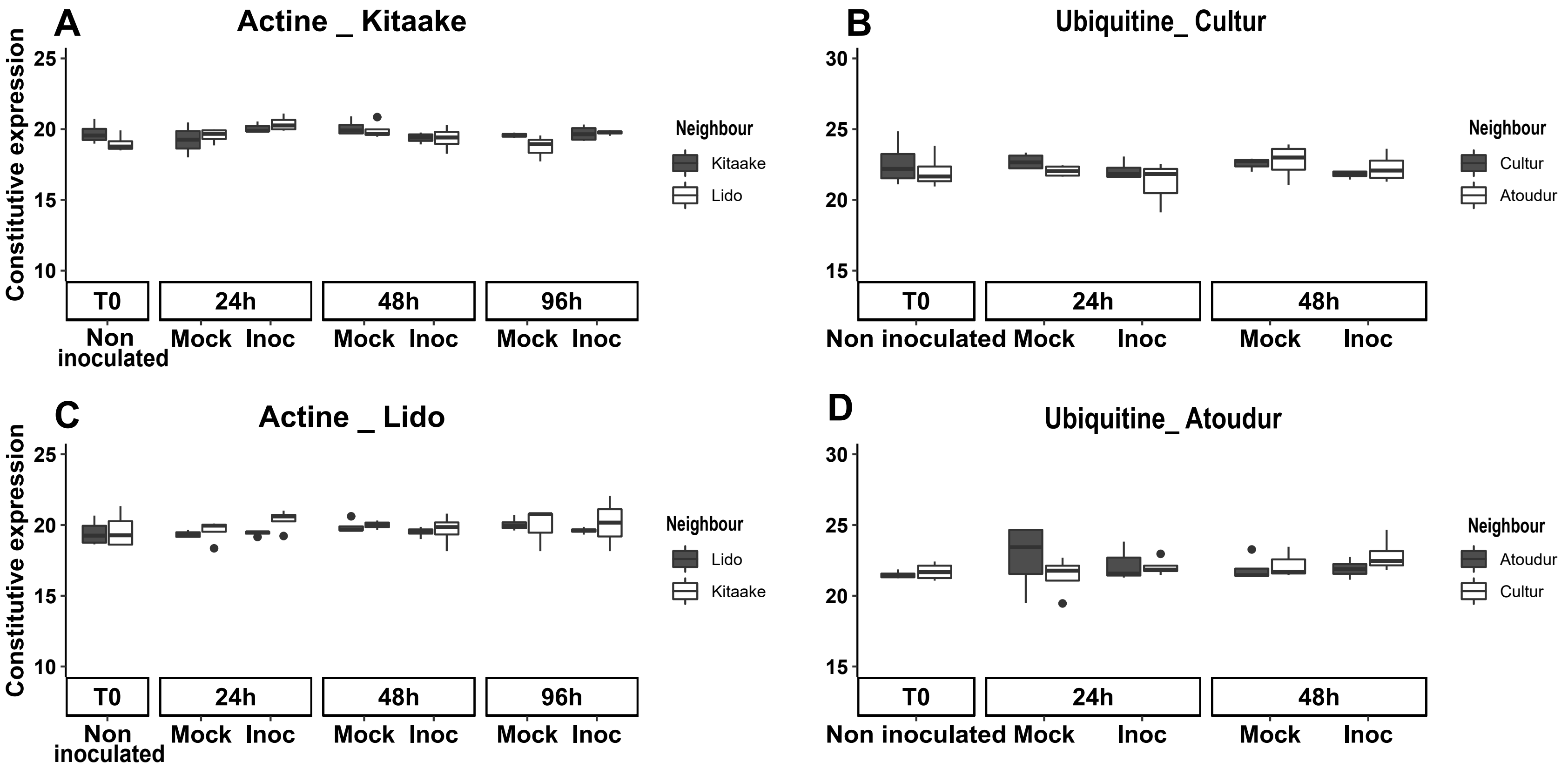

Fig. S2 Expression of housekeeping genes

Gene expression was meas ured leaves of plants grown in pure (grey bars) or mixture conditions (white bars) in the same conditions presented in the Fig.5 et Supplementary Fig.S5. For rice, KIT (A) or LID (C) were grown with itself or in the presence of conspecific LID and KIT respectively. For wheat, CUL (B) or ATO (D) were grown with itself or in the pre sence of the conspecific neighbo ur ATO or CUL.The expression of each of housekeeping genes , actin (Os03g50890) and ubiquitin (CD921597) for rice and wheat respectively, was measured by Quantitative RT- PCR. The original CT obtained from 1:10 cDNA prepared with 5ug of RNA are shown. The mean +/- SE of at least 4 replicates is shown. Statistical groups were calculated by Tukey HSD test and no significant differences were found between groups (neighbour, time or inoculation) for each genotype.

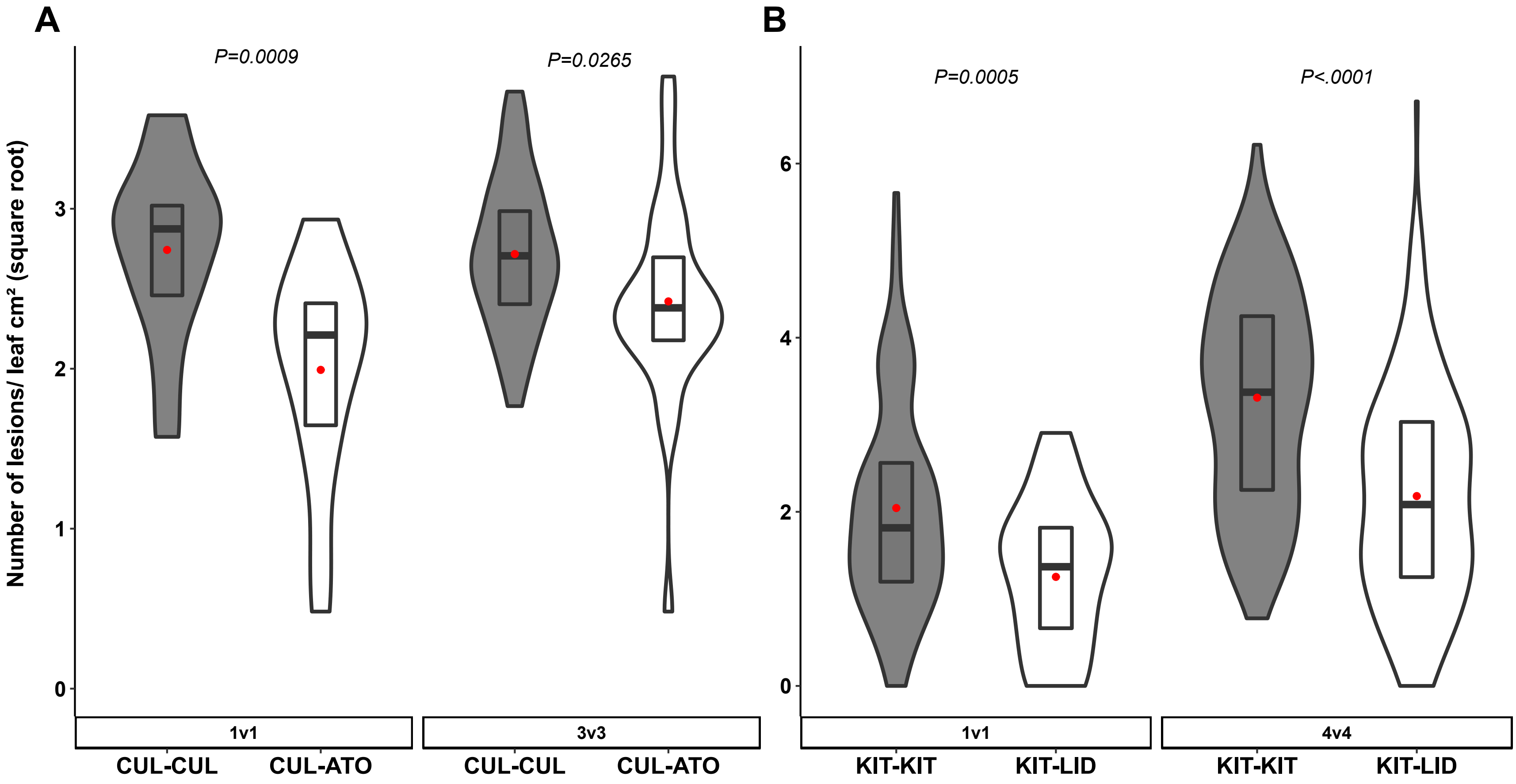

Fig. S3 Evaluation of intra-genotypic and inter-genotypic contributions to plant-plant interactions.

Mixture models or wheat (A) and rice (B) were put in conditions allowing inter-genotypic interactions (1v1: 1 focal plant and 1 neighbour) or intra- and inter genotypic interactions (3v3: 3 focal and 3 neighbour plants and 4v4: 4 focal and 4 neighbour plants). The volume of soil in 1v1 and 3v3 or 4v4 experiment was adjusted to maintain similar plant density. Mixtures and their controls were inoculated with the pathogen *P.triticina* for wheat and *M. oryaze* for rice. For wheat, the focal plant CULTUR was grown with itself (CUL-CUL) or in the presence of ATOUDUR conspecific neighbour (CUL-ATO). For rice, the focal plant KITAAKE was grown in the presence of itself (KIT-KIT) or in the presence of the conspecific neighbour LIDO (KIT-LID). Violin plots are representing the distribution of the data, the red dots represent the mean of the dataset. Each violin plots represented at least n=42 (4v4) or n=18 (1v1) plants for rice and n=36 (3v3) or n=18 (1v1) plants for wheat. At least 3 independent different experiments of 4 or 6 replicates each for wheat and rice respectively are shown. Statistical p values were determined according to ANOVA, followed by a Dunnett test on the linear model described in Methods.

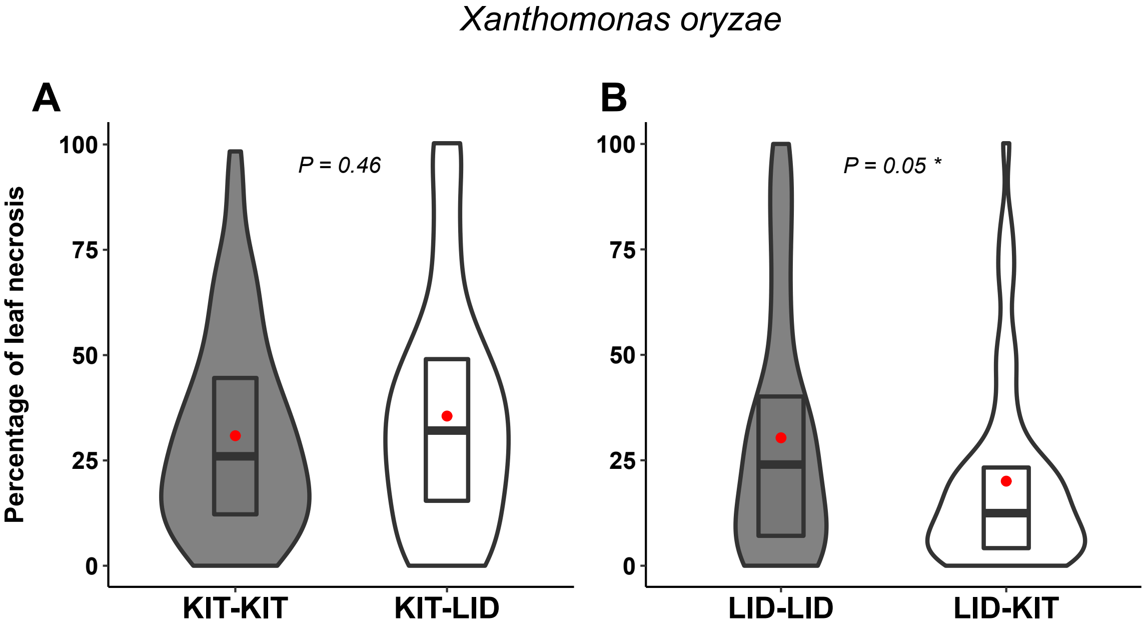

Fig. S4 Response of selected intra-specific mixtures of rice on disease susceptibility to *Xanthomonas oryzae.*

Rice plants in the mixture model between KITAAKE and LIDO were inoculated with the bacterial pathogen *Xanthomonas oryzae* pv. *oryzae* and lesion size was measured on each genotype in the presence of itself (KIT-KIT and LID-LID) or in the presence of its conspecific neighbour (KIT-LID and LID-KIT). Violin plots are representing the distribution of the data, the red dots represent the mean of the dataset. Each violin plots represented at least n=42 plants. At least 3 different experiments of 6 replicates each are shown. Statistical p values were determined according to ANOVA, on the linear model described in Methods.

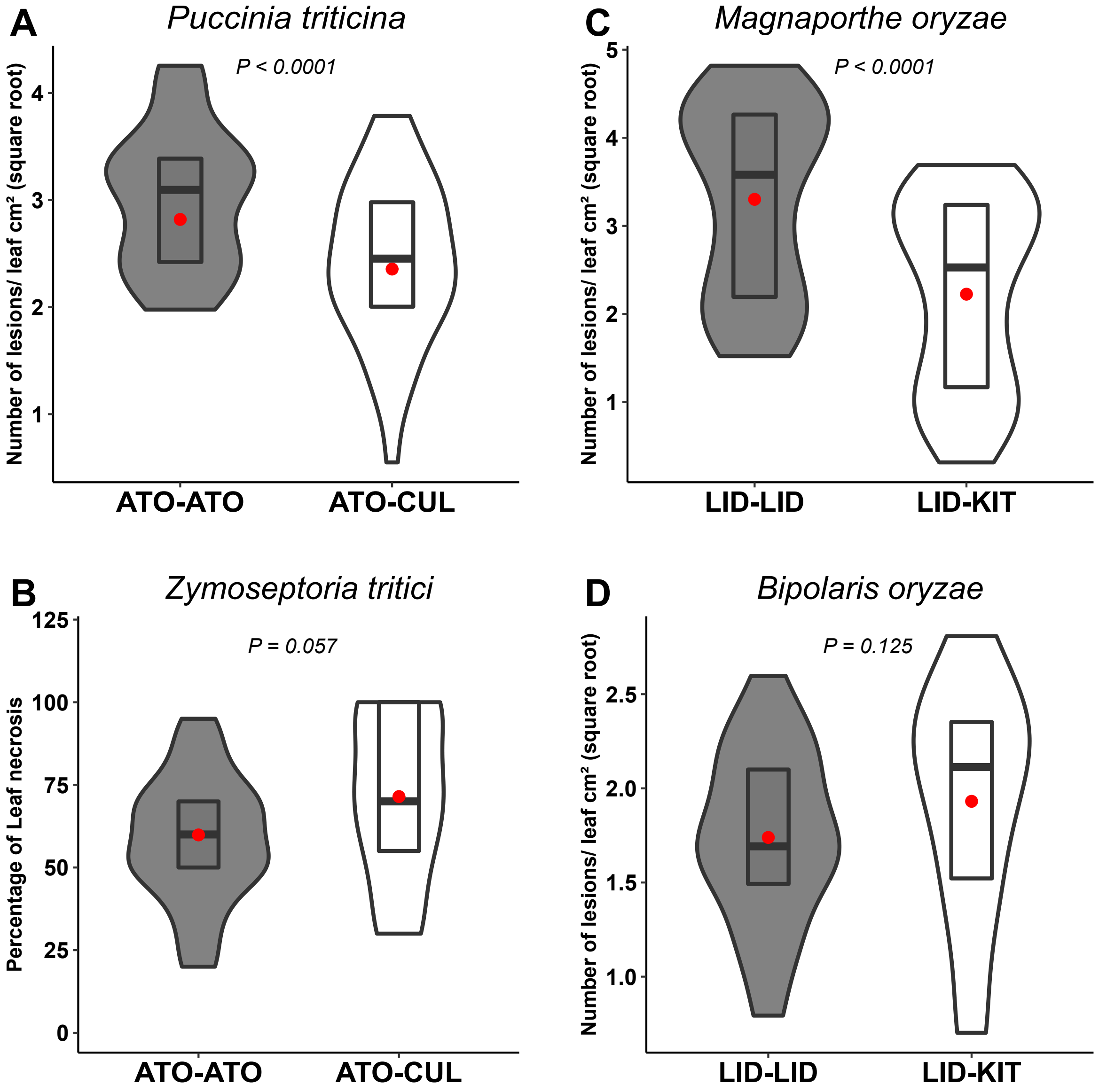

Fig. S5. Effects of different pathogen lifestyles on responses of disease susceptibility in selected intra-specific mixtures in rice and wheat.

Mixture models and their pure control were inoculated with the fungal pathogen indicated above each graph. For wheat (A and B), the focal plant ATOUDUR was grown with itself (ATO-ATO) or in the presence of the conspecific neighbour CULTUR (ATO-CUL). For rice (C and D), the focal plant Lido was grown in the presence of itself (LID-LID) or in the presence of the conspecific neighbour KITAAKE (LID-KIT). Violin plots are representing the distribution of the data, the red dots represent the mean of the dataset. Each violin plots represented at least n=42 plants for rice and n=36 plants for wheat. At least 3 different experiments of 4 or 6 replicates each for wheat and rice respectively are shown. Statistical p values were determined according to ANOVA, on the linear model presented in Methods.

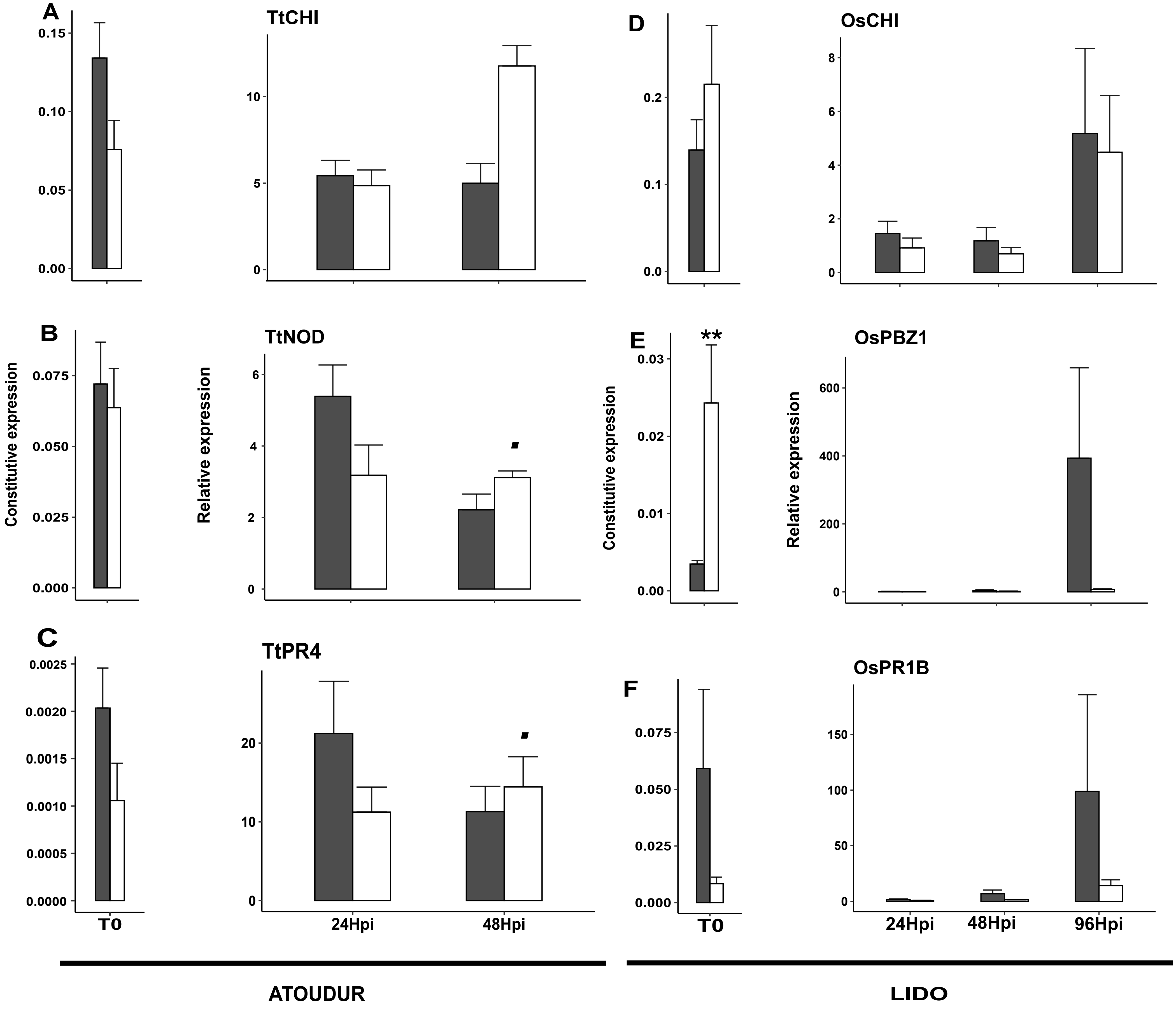

Fig. S6. Expression of immunity-related genes in the wheat ATO genotype and the rice LID genotype when they were the focal plants (reciprocal data for Fig. 5).

Gene expression was measured leaves of plants grown in Pure (grey bars) or mixtures (white bars). For wheat, Atoudur was grown with itself or in the presence of the conspecific neighbor Cultur (A, B and C). For rice, Lido was grown with itself or in the presence of conspecific Kitaake (D, E and F). For each graph, the name of the defense gene is indicated (*Tt* genes for wheat and *Os* genes for rice). Gene expression was measured by before infection (T0) and at different hours post inoculation (hpi) with *P. triticina* for wheat (24 and 48 hpi) and *M.oryzae* for rice (24, 48 and 96 hpi). The expression of each defense marker gene measured by Quantitative RT -PCR was normalized with actin and ubiquitin for rice and wheat respectively. For the expression data before inoculation (T0), the constitutive expression is shown while for time points after inoculation, a ratio of expression between inoculated and non-inoculated conditions is shown. The mean +/- SE of at least 6 replicates is shown. The statistical differences, as estimated by Wilcoxon tests, between conditions are shown for each time point (. : p < 0,1 ; * : p < 0,05 ;** : p < 0,01). The data corresponding to the reciprocal genotypes considered as focal is provided in Fig.5.

Table S1. Summary of the experiences and expected outcomes

Treatment	Outcomes awaited	Pathogen	Plant species	Focal tested	Neighbour
Screening experience (**Fig.1**)	Identification of pairs	Magnaporthe oryzae	Rice	KITAAKE	9 different temperate japonica
		Pucina triticina	wheat	CULTUR	9 different Durum wheat
Inter genotypic experience (1v1) (**Fig.S2**)	Comparaision of intra and inter genotypic interaction	Magnaporthe oryzae	Rice	KITAAKE	LIDO
		Pucina triticina	wheat	CULTUR	ATOUDUR
Disease assesment (**Fig.2**, **Fig.S3,S4**)	Impact of mixture on disease susceptibility	Magnaporthe oryzae	Rice	KITAAKE	LIDO
				LIDO	KITAAKE
		Bipolaris oryzae		KITAAKE	LIDO
				LIDO	KITAAKE
		Xanthomonas oryzae		KITAAKE	LIDO
				LIDO	KITAAKE
		Pucina triticina	wheat	CULTUR	ATOUDUR
				ATOUDUR	CULTUR
		Zymoseptoria tritici		CULTUR	ATOUDUR
				ATOUDUR	CULTUR
Soil sterilization (**Fig.3**)	Implication of the root microbiota	Magnaporthe oryzae	Rice	KITAAKE	LIDO
		Pucina triticina	wheat	CULTUR	ATOUDUR
Porus soil separation (**Fig.3**)	Requirement of root contact	Magnaporthe oryzae	Rice	KITAAKE	LIDO
		Pucina triticina	wheat	CULTUR	ATOUDUR
Plastic soil separation (**Fig.3**)	Implication of soil compartment	Magnaporthe oryzae	Rice	KITAAKE	LIDO
		Pucina triticina	wheat	CULTUR	ATOUDUR
Neighbour unifected (**Fig.4**)	Requirement for infected or uninfected neighbour	Magnaporthe oryzae	Rice	KITAAKE	LIDO
		Pucina triticina	wheat	CULTUR	ATOUDUR
Neighbour inoculated and covered after inoculation (**Fig.4**)	Requirement of aerial contact communication	Magnaporthe oryzae	Rice	KITAAKE	LIDO
		Pucina triticina	wheat	CULTUR	ATOUDUR
Transcriptomic analysis (**Fig.5**, **Fig.S5**)	Expression of defense gene in mixture	Magnaporthe oryzae	Rice	KITAAKE	LIDO
				LIDO	KITAAKE
		Pucina triticina	wheat	CULTUR	ATOUDUR
				ATOUDUR	CULTUR

Table S2. List of the durum wheat genotypes used in this study

Name	Origin	Abreviation
ALEXIS	FRANCE	ALE
ATOUDUR	FRANCE	ATO
ARGELES	FRANCE	ARG
CULTUR	FRANCE	CUL
JANEIRO	FRANCE	JAN
LIBERDUR	FRANCE	LIB
GALADUR	FRANCE	GAL
NEODUR	FRANCE	NEO
OBELIX	FRANCE	OBE
SCULPTUR	FRANCE	SCU

Table S3. List of the temperate japonica rice genotypes used in this study

Name	Origin	Abreviation
BOMBILLA	Italy	BOM
CARINA	Italy	CAR
GRITNA	Italy	GRI
KING	Italy	KIN
LIDO	Italy	LID
LUXOR	Italy	LUX
MARATELLI	Italy	MAR
NIPPONBARE	Japan	NIP
KITAAKE	Japan	KIT
SALVO	Italy	SAL

Table S4. List of the wheat genes used in this study

Gene name	Gene ID	Primer sequence
Ubiquitin	CD921597	CTGGCGAGGATATGTTCCAT<br>TCGGATGGAAGACCTTTGTC
TtNOD	Ta.27299.1.S1_x_at	GCCGGTTGCACGAGTAAA<br>TTACGAAAAGCGGAAGAGGA
TtPR4	Ta.9226.1.S1_at	ATAAAAGGCTAGCTAAAAGTAAGTTGA<br>TTTGAATAGATAACCCGTTTGG
TtCHI	Ta.2784.1.A1_at	TGCGCTACTGCAGCATACTC<br>ACCATACGAATCAACTGATCACA

Table S5. List of the rice genes used in this study

Gene name	Gene ID	Primer sequence
Actine	Os03g50890	GCGTGGACAAAGTTTTCAACCG<br>TCTGGTACCCTCATCAGGCATC
OsPBZ1	Os12g36880	CCTGCCGAATACGCCTAAGATG<br>AGAACACATTCAGACTTGCCTCTC
OsPR1B	Os01g28450	CGAGAAGAGCGACTACGACTAC<br>GCCTCTGTCCGACGAAGTTG
OsCHI	Os07g35560	TTAACGGCGCTGCTACCATT<br>TCCCATCCTCTTACTGCCGA
